# Three-dimensional functional unit analysis of hemifacial microsomia mandible—a preliminary report

**DOI:** 10.1186/s40902-015-0027-z

**Published:** 2015-09-16

**Authors:** Ji Wook Choi, Byung Hoon Kim, Hyung Soo Kim, Tae Hoon Yu, Bong Chul Kim, Sang-Hwy Lee

**Affiliations:** 1 Department of Oral and Maxillofacial Surgery, College of Dentistry, Yonsei University, 50 Yonsei-ro Seodaemun-Gu, Seoul, 120-752 Korea; 2 Department of Oral and Maxillofacial Surgery, Daejeon Dental Hospital, College of Dentistry, Wonkwang University, Daejeon, Korea

**Keywords:** Hemifacial microsomia, Functional unit, Mandible, Three-dimensional, Computerized tomography

## Abstract

**Background:**

The aim of this study was to present three-dimensional (3D) structural characteristics of the mandible in the hemifacial microsomia. The mandible has six distinct functional units, and its architecture is the sum of balanced growth of each functional unit and surrounding matrix.

**Methods:**

In order to characterize the mandibular 3D architecture of hemifacial microsomia, we analyzed the mandibular functional units of four hemifacial microsomia patients using the 3D reconstructed computed tomography (CT) images. And we compared the functional unit size between affected and non-affected side.

**Results:**

The length of condyle and angle showed significant differences between affected and non-affected sides. However, the length of mandibular body showed insignificant differences. The size differences between affected and non-affected side were observed at the condyle, angle, and body in descending order.

**Conclusions:**

This preliminary study suggests that the main etiopathogenic units are condyle and angle in the hemifacial microsomia mandible. Further investigation with the increased number of subjects will be helpful to establish treatment modality by etiopathogenic targeting of hemifacial microsomia.

## Background

Hemifacial microsomia is a congenital disease of craniofacial region. This disease occurs in 1/3500–1/5600 of frequency to make it the second most common congenital deformity next to cleft lip and palate at the craniofacial region [1]. It is called as such hemifacial microsomia because it occurs mainly on one side of the face and is manifested as the small jaw. But it can be manifested at both sides of the face simultaneously in 10–15 % of patients [2, 3] that it can be named as the craniofacial microsomia. It can be also called as the first pharyngeal arch syndrome due to its characteristics of occurrence at the first and second pharyngeal-originated facial structures [4]. They include the mandible, maxilla, orbit, external and middle ear, craniofacial nerve, and facial soft tissue [5, 6].

Hemifacial microsomia can present the variable signs and symptoms, ranging from the slight asymmetry of face to the complete absence of one ear, small ipsilateral face, facial nerve palsy, and the cleft of the mouth corner [7]. Different classification systems have been suggested to accommodate these variable clinical manifestations [8–10]. OMENS system is one of such classification, which represents the abbreviation of first letter of orbit, mandible, ear, nerve, and soft tissue [9]. This classification method was reported more recently than other systems did; it addresses the five major manifestations of hemifacial microsomia and allows each to be graded separately according to severity. Another classification method is the Pruzansky classification that has been used more frequently because it has more detailed grading of mandibular shape [10] (Table 1).

**Table 1:** Classification of hemifacial microsomia by Pruzansky (1969)

Type	Features
I	All the elements of the mandible exist
	Various degree of hypoplasia of mandible
	Decrease of cartilage and articular cavity size of the temporomandibular joint
	Normal hinge movement with mouth opening limitation
	All masseteric muscles are in low volume but in normal range
IIA	Abnormal shape of articular structure
	The head of the condyle is cone shape and anterior than normal position
	The coronoid process and gonial angle are normal
	Temporomandibular joint is able to move in hinge axis but translation is impossible
	All masseteric muscles exist but are in hypotrophy
II B	No articulation exists between the condyle and temporal bone
	Various sizes of coronoid process
	Possible abnormal position of the condyle
	Various defects of pterygoid and masseter muscles
	No adhesion of lateral pterygoid muscles to the mandible
III	Deficiency of the mandibular ramus
	Significant hypotrophy of masseteric muscles
	No adhesion of lateral pterygoid and temporalis muscles to the mandible

Treatment of hemifacial microsomia includes orthodontic treatment and surgical correction. The treatment timing and method varies according to patient’s age, degree of facial deformity, degree of skeletal deformity, and the surgical treatment strategy. The costochondral grafting was performed as one of the main treatment procedures from 1970s to 1990s [11]. Since the introduction of distraction osteogenesis on craniofacial region in 1995, it replaced the grafting and has become one of the most popular surgical treatment options so far [2, 12].

The functional unit and its analysis is a way of geometrical understanding for the biological structure [13]. The functional units of mandible are reported to be composed of condyle, coronoid, body, angle, symphysis, and dentoalveolus [13, 14], which are known to be growing independently. Thus, we can presume that the characteristics of functional units in hemifacial microsomia can be different at its affected part or unit and the degree of involvement during the development and growth.

In order to attain the ideal treatment of hemifacial microsomia including the mandibular distraction osteogenesis, we should locate the region of the affected mandibular structure, which had been abnormally developed and grown. Then, we will be able to focus on this part to elongate or correct the mandible. But unfortunately, the decision of region for operation has been mainly based on the gross evaluation of morphology in addition to the surgeon’s experiences. If we evaluate the structural characteristics of functional unit for hemifacial microsomia, we can anticipate the treatment, such as distraction osteogenesis, to be more effective, easier with better result. Therefore, in this study, we performed the three-dimensional morphologic analysis of hemifacial microsomia mandible by functional unit analysis to attain the appropriate diagnosis and treatment plan, especially with the decision of the site for distraction osteogenesis.

## Methods

Four subjects aged 5–18 years were diagnosed with unilateral hemifacial microsomia at the Department of Oral and Maxillofacial Surgery, Yonsei University Dental Hospital. Their computed tomography (CT) scans were obtained with the high-speed advantage CT scanner (GE Medical System Milwaukee, USA) used with high-resolution bone algorithm (200 mA, 120 kV) at 1 s, 1 mm slice thickness, and 512 × 512 pixel reconstruction matrix. Written informed consent was obtained from the subject for the publication of CT images.

**Fig. 1:**
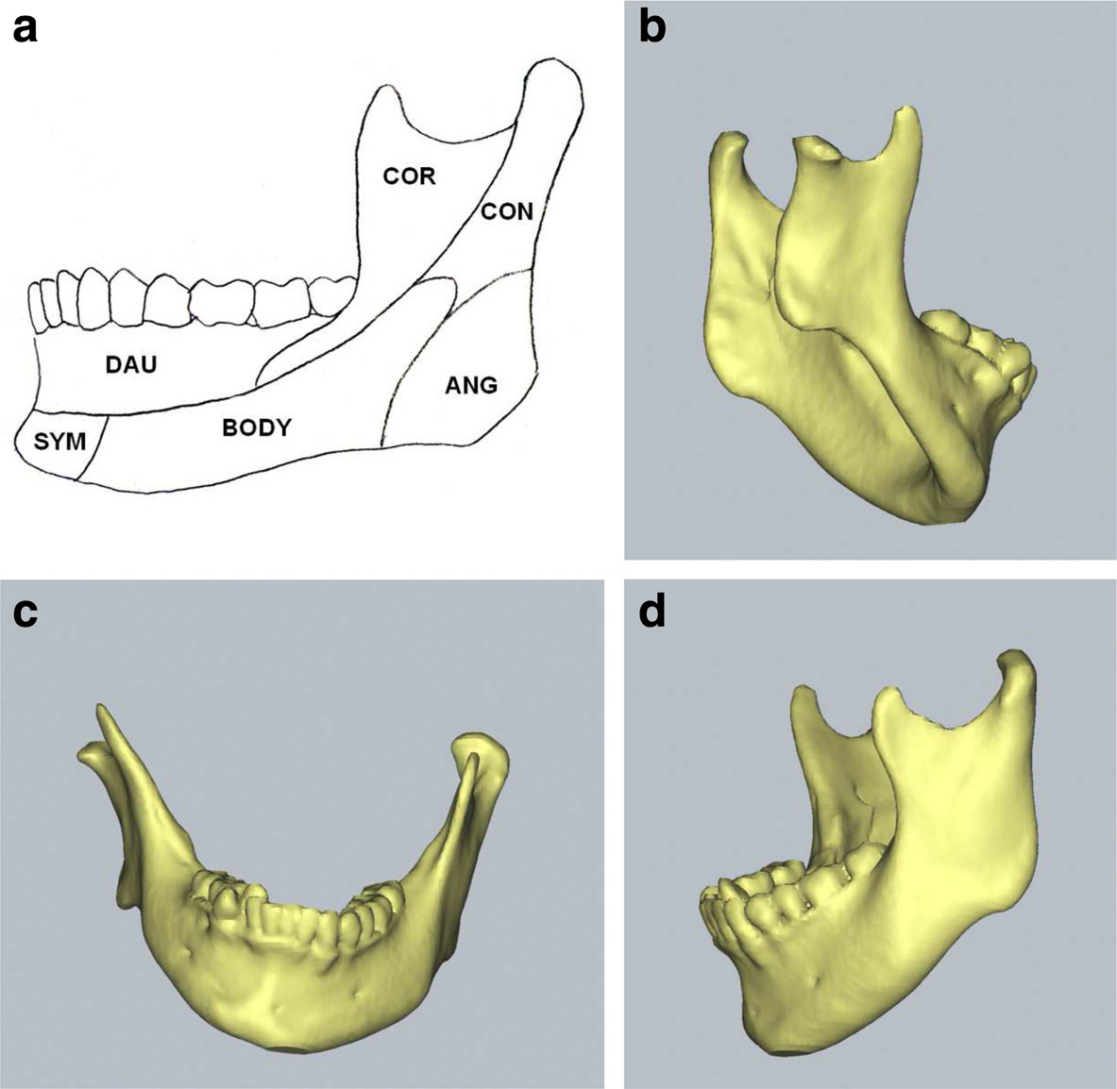
Mandibular functional unit and three-dimensional computerized tomographic images of hemifacial microsomia mandible with Pruzansky’s type I deformity. **a** Mandibular functional units. **b** Affected side of hemifacial microsomia mandible. **c** Frontal view. **d** Non-affected side

**Fig. 2:**
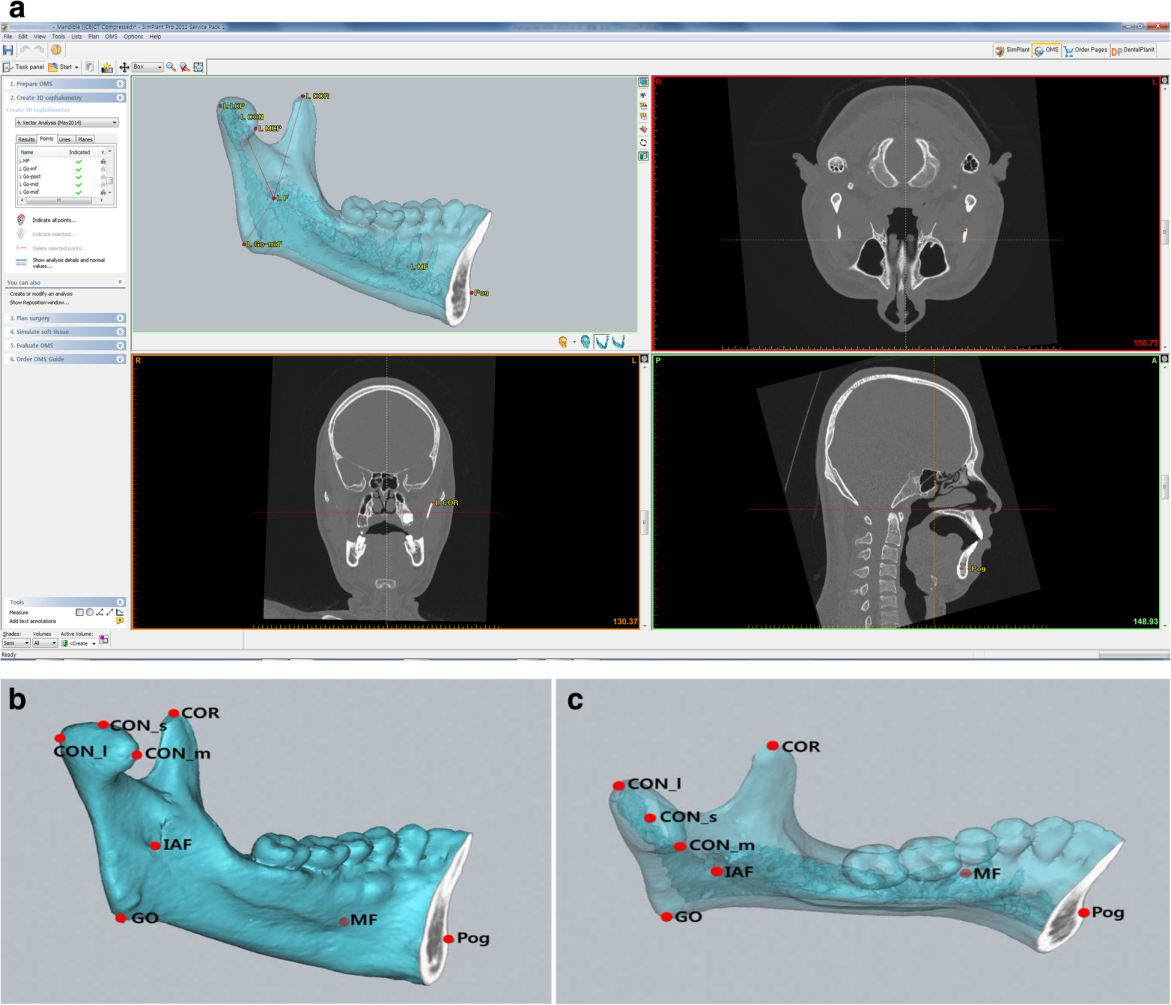
Mandibular reference points for the mandibular functional unit analysis on the normal mandibular structure. **a** A screen capture image of the software used in this study (SimPlant, version 14.0, Materialise NV, Leuven, Belgium). **b** Lateral view of hemifacial microsomia mandible with reference points. **c** Occlusal view

Reformatted three-dimensional (3D) images were created from the CT scan data, and the measurements of the mandibular functional unit length were performed using SimPlant software (version 14.0, Materialise NV, Leuven, Belgium) (Fig. 1). The detailed measurement method was followed by our previous report [15]. We divided the mandible into six functional units: condyle, coronoid, body, angle, symphysis, and dentoalveolus, according to Moss and Simon’s suggestion [13], after defining the reference points as follows (Fig. 2):

1. Mandibular foramen (IAF): The most inferior point on the mental foramen.
2. Mental foramen (MF): The entrance point of the mental foramen.
3. Condyle_superior (CON_s): The most superior point of the condyle.
4. Condyle_lateral (CON_l): The most lateral point of the condyle.
5. Condyle_medial (CON_m): The most medial point of the condyle.
6. Coronoid (COR): The most superior point of the coronoid process.
7. Gonion (GO): The most inferior, posterior, and lateral point on the external angle of the mandible.
8. Pogonion (Pog): The most anterior point of the mandible suture.

**Fig. 3:**
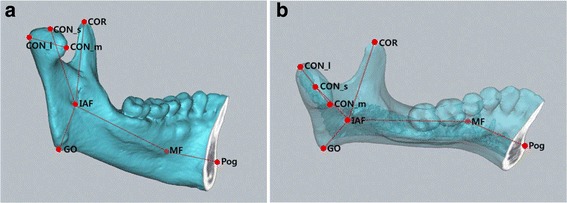
Mandibular functional unit measurement with reference points on the normal mandibular structure. **a** Lateral view of hemifacial microsomia mandible. **b** Occlusal view

The length of each functional unit was set as the distance between two reference points (Fig. 3). The condyle length was defined as the distance between IAF and CON_s; the coronoid length was defined as the distance between IAF and COR. The angle length was defined as the distance between IAF and Go, the body length was defined as the distance between IAF and MF, and the symphysis length was defined as the distance between MF and Pog. Additionally, the condylar head length was defined as the distance between the CON_m and CON_l.

## Results

All four subjects of hemifacial microsomia were classified based on Pruzansky’s classification [10]. Three subjects were classified as type I and one remaining as type IIb. The size of the functional units of the affected and non-affected side in length was measured and compared (Table 2). All of them showed the unilateral involvement, without any bilateral defect.

**Table 2:** Analysis of mandibular functional units

	Subject	Condyle	Coronoid	Angle	Body	Symphysis	Condyle head
Non-affected side (mm)	A	45.16	42.43	33.66	55.56	27.49	24.92
	B	42.94	32.43	15.79	43.85	27.33	16.45
	C	34.82	27.97	21.38	52.45	27.11	16.77
	D	36.61	33.44	19.03	43.2	26.14	13.18
Affected side (mm)	A	31.8	36.34	26.24	44.59	24.44	22.7
	B	32.67	31.98	14.66	42.77	29.67	16.23
	C	29.23	30.04	19.61	51.61	24.29	13.3
	D	26.77	31.51	18.27	41.72	27.4	10.76
Difference (mm)	A	13.36	6.09	7.42	10.97	3.05	2.22
	B	10.27	0.45	1.13	1.08	−2.34	0.22
	C	5.59	−2.07	1.77	0.84	2.82	3.47
	D	9.84	1.93	0.76	1.48	−1.26	2.42
Difference (%)	A	29.58	14.35	22.04	19.74	11.09	8.90
	B	23.91	1.39	7.16	2.46	−8.56	1.34
	C	16.05	−7.4	8.28	1.6	10.4	20.7
	D	26.88	5.77	4	3.43	−4.82	18.36
Relative length (%)	A	70.4	85.65	77.96	80.38	80.91	91.09
	B	76.08	98.61	92.84	97.54	108.56	98.66
	C	83.94	107.4	91.72	98.39	89.6	79.3
	D	73.12	94.22	96	96.57	104.82	81.64

Difference (%) = (difference between affected and non-affected side / length of non-affected side) X 100

Relative length (%) = (length of affected side / length of non-affected side) X 100

The size of the condyle unit showed the greatest discrepancy of length between the affected and non-affected side. The condyle length of the affected side was 32.7–26.8 mm (average 30.1 mm), while the non-affected control side was 45.2–34.8 mm (average 39.9 mm). And the difference between the size of affected and non-affected side ranged from 13.4 to 5.6 mm to make the size of affected side be 70–84 % of the non-affected size. The subject with Pruzansky’s type IIb showed the greatest difference of condylar unit size between the affected and non-affected side.

The angle length was the second greatest difference after the condylar unit, with the size differences (average 2.8 mm) ranging from 7.4 mm (22 %) to 0.8 mm (4 %). The subject with Pruzansky’s type IIb again showed the greatest difference in angular unit size between the affected and non-affected side (7.4 mm).

On the other hand, the mediolateral length of condylar head showed the least differences (3.5–0.2 mm, average 2.1 mm). In addition, the differences of the body length were relatively small with the average difference of 3.6 mm (11.0–0.84 mm) to reach the size of affected side to be 93 % (80–98.4 %) of the non-affected side.

## Discussion

Though it is known well that hemifacial microsomia is a congenital malformation in most cases, there are only a few presumptions that it can be caused by natural mutation rather than heredity and/or by drugs like thalidomide, primidone, or retinoic acid [5]. Some other authors suggested that hemifacial microsomia can be caused by the stapedial artery hematoma [1] or abnormal neuroectodermal cell migration during embryogenesis [6]. The former theory explains that the hemorrhage from the stapedial artery produces hematoma to induce the pressure around the first and second pharyngeal arch. Thus, the size and shape of hematoma can be related to the phenotypic variability. The latter theory proposes that retinoic acid changes the pattern of migration and/or the distribution of neural crest cells, which finally incur the deformity of the facial tissues from pharyngeal arches. However, the exact pathogenesis for hemifacial microsomia is still unclear even with these hypotheses.

There is also a controversy about the growth pattern of hemifacial microsomia. Some authors reported that patients have mild facial abnormality at birth, but their asymmetry becomes more distinct as the non-affected side grows faster than affected side does [11]. However, others suggested that the degree of facial deformity is not accelerated during growth [16]. Thus, it is not clear again about the growth pattern of hemifacial microsomia, as in its pathogenesis [6]. We hope our analysis with functional unit in large sample size can be of help to support them.

Variety of clinical and supplementary data, including the facial photos, plaster dental models, and radiographic images, can be used for diagnosis of hemifacial microsomia. Especially, the two-dimensional cephalometric radiography has been the main diagnostic tool. However, it has inevitable limitations such as the image expansion and distortion and the blurring of superimposed anatomical structures. So there are difficulties in precise diagnosis and treatment planning of three-dimensional craniofacial structures [17–19].

Since the first introduction of CT in 1979, three-dimensional CT (3D CT) became a major imaging tool for craniofacial evaluation and treatment planning. Even though 3D CT needs high-dose radiation and expensive cost, it can allow us to observe the craniofacial structure at the various perspectives and to analyze 3D length and angle more precisely than two-dimensional cephalometric radiography does [18, 19]. Furthermore, there are no image distortions, and the deep structures can be directly observed by controlling images. Nowadays, 3D imaging software for CT can be easily accessed with the personal computer environment [17], and the development of high-quality CT machine such as the multi-detector CT and the cone-beam CT make it possible to acquire thin sliced CT image (being less than 0.5 mm) and to reduce radiation dose with the special low-dose protocols. Therefore, the 3D CT is expected to be more popular for craniofacial imaging in the future [15].

The 3D imaging technology for craniofacial deformity is developing rapidly up to the level, which can measure the length and angle for anatomic structure at the complex craniofacial region, make a 3D simulational operation, and predict the outcome after the simulational surgery [17–19]. But the 3D technology for diagnosis and treatment planning has not been applied enough to the field of hemifacial microsomia and congenital dysmorphosis. We will need more works for the 3D understanding of biological structures, the confirmation of etiopathogenic mechanism and region, and the simulational planning to reconstruct the craniofacial structure to be normal.

The mandible is the main anatomic structure on the lower part of craniofacial region, and it can have a strong influence on the development of malocclusion and craniofacial deformity. At 10th week of human embryonic development, the membranous bone begins to be formed near the mental foramen after Meckel’s cartilage development and calcification [20]. It is progressed along the inferior alveolar nerve to mandibular foramen. Then, a primitive mandible is completely formed with the additional development of secondary cartilage at the condyle, coronoid process, and symphysis region [14]. After the fetal isometric growth and its birth, the mandibular growth is attained by the longitudinal growth, similar to that of the long bones, at the condylar region and also by the superficial apposition and resorption of bones [14].

Based on this process of the mandibular development and growth, Moss and Simon assorted functional units using functional matrix theory [13]. Precious and Delaire additionally proposed that the mandibular growth is the sum of independent growth of each mandibular functional unit [21]. Based on these theories, the mandible can be divided into the unit of the condyle, coronoid, body, angle, symphysis, and dentoalveolus. And the functional matrix, best exampled by the masseter muscle, can affect the growth of these units while being affected reciprocally.

Distraction osteogenesis is one of the ideal treatment strategies for hemifacial microsomia, as described previously. In order to apply this treatment strategy to the treatment of hemifacial microsomia, it is necessary to understand the long-term effectiveness of this distraction modality. On a report written by Meazzini et al. [22], the distraction was performed at the ramus of the mandible for eight patients with type I and II hemifacial microsomia at an average age of 5.6 years old. Five years postoperatively, the ratio between affected and non-affected rami returned to 77 % of the correction obtained by the distraction. Huishinga-Fisher et al. [23] also reported that the distraction osteogenesis was performed in eight children and about 50 % of cases seemed to have relapse, which occurred 1 year after distraction osteogenesis. And these relapses seemed to progress up to 3 years after distraction osteogenesis.

Thus, the decision about the optimal timing of distraction osteogenesis should consider the mandibular growth pattern and the effect of distraction osteogenesis to the mandibular growth pattern. If facial asymmetry and deformity in hemifacial microsomia become worse during the growth period, the early application of distraction osteogenesis will be necessary to prevent secondary deformity [11]. However, if the degree of deformity is not worsened during the growth period, the surgical correction should be delayed until the growth is finished [6]. There are few studies about the growth pattern of hemifacial microsomia to acquire the conclusion about this. But Grayson reported in his long-term follow-up study that the vertical bone growth in hemifacial microsomia is not definite after distraction osteogenesis [24]. Moreover, Marquez reported that the growth ratio of affected side is reduced after distraction osteogenesis [25]. Considering all these reports, the early stage treatment seems to have less advantage in terms of long-term treatment effect.

It is also controversial at which the distraction osteogenesis can be applied. Mommaerts and Nagy suggested that the results of distraction osteogenesis at the mandibular body are more stable, and the body part may be more important than the ramus does [26]. But Kusnoto et al.’s report does not allow clear conclusion about the vertical stability of the distraction osteogenesis-induced new bone between the mandibular body and ramus [27].

The functional treatment goal for hemifacial microsomia is to restore the normal function and structure and to induce the normal growth by recovering the affected mandibular functional unit. So we tried in this study the analyses of hemifacial microsomia for 3D mandibular shape to find the affected functional unit, at which we can apply the distraction osteogenesis. Though the sample size is too much limited, we could obtain the result saying that the size differences between affected and non-affected side were observed at the condyle, angle, and body in descending order. So we can assume that the ramus, especially the condylar and angular unit, is the most etiopathogenic or affected area in hemifacial microsomia. Particularly, the lack of length at the affected the condyle unit reached about 70 % as compared with that of the non-affected side. Based on these results, the affected ramus may be treated by distraction osteogenesis to lengthen the short condyle unit and surrounding muscles. Additional treatment to the angular unit also needs to be considered because the insufficient size of mandibular angle may not be resolved by distraction osteogenesis alone. The distraction at the angular region or the free bone graft may be a possible candidate solution.

Meanwhile, the size difference of mandibular body unit at the affected and non-affected side was not as evident as had been expected in this study. The mandibular body unit is intimately related to the development of the inferior alveolar neurovascular bundle during the developmental period. Neiva et al. measured the length of inferior alveolar canal with 3D CT for hemifacial microsomia [28]. And they found no significant difference of the bony canal length between the affected and non-affected side of Pruzansky’s type I group subjects, while those of Pruzansky’s type II to be significantly different. According to their finding, we could assume that the insignificant difference of body length might be related to the small-sized sample of this study. Three subjects out of four belonged to Pruzansky’s type I deformity in this study. So we cannot expect the result of this study to be applied to the general mandibular shape of hemifacial microsomia, and more studies for this point will be necessary in the future. Furthermore, the individualized analysis of each hemifacial microsomia has to be performed to make customized treatment planning for individual patients. Nevertheless, the mandibular functional unit analysis to find the major etiopathogenic area can be a useful diagnosis tool for hemifacial microsomia.

## Conclusions

The functional unit analysis of hemifacial microsomia mandible was performed on the 3D CT images.

The condyle unit consistently showed the greatest size difference between the affected and non-affected side of mandible. And the size of the affected side condylar unit reached about 70 % of the non-affected side.

And the size difference of mandibular angle unit was the second greatest, when the affected and non-affected angular unit are compared.

The size difference at the mandibular body unit was relatively small, and the mediolateral length of the condylar head showed the least differences.

These findings suggest that the main etiopathogenic units of the hemifacial microsomia mandible may be the condylar and angular functional unit.
